# Role of nanomedicine in COVID-19 therapeutics

**DOI:** 10.2217/nnm-2021-0358

**Published:** 2022-01-11

**Authors:** Smriti Sharma

**Affiliations:** ^1^Molecular Modeling and Drug Design Laboratory, Department of Chemistry, Miranda House, University of Delhi, New Delhi, 110007, India

**Keywords:** coronavirus, COVID-19, COVID-19 therapeutics, nanodrugs, nanoformulations, nanomedicine, nanoparticles, nanotechnology, SARS-CoV-2, vaccines

The last 2 years have seen an unprecedented jump in global collaborative efforts to find a cure for COVID-19. Nanomedicine is fast providing solutions to the problem of designing new vaccines for coronavirus because nanomaterials are perfect vehicles for delivering antigens, and they can mimic the structure of the virus and play the role of adjuvant perfectly [1]. A testament to the promise of nanomedicine for curing COVID-19 is the use of lipid nanoparticles (NPs) for delivering the mRNA vaccine, which was the first vaccine that was sent for clinical trials [2]. The success in managing a pandemic like COVID-19 depends on four factors: the speed at which a new vaccine is designed, scalability of manufacture, effective global distribution and successful administration of the vaccine to the population. This is fraught with many logistical roadblocks, such as nonavailability of high-quality cold chains, especially in economically weaker countries, and the duration for which the vaccine remains efficacious after administration [3].

Nanomedicine is providing some innovative solutions to these demanding challenges by aiding in the upscaling, stability, distribution, portability and development of technologies to help with the administration of COVID-19 vaccines. For instance, opto-, electro- and magnetobiosensors based on nanotechnology are used for the detection of SARS-CoV-2 virus at very low concentrations [4,5]. These miniaturized sensors can be integrated with smartphone devices and can aid in point-of-care diagnosis of COVID-19. In addition, these smartphone-enabled nanobiosensors can be coupled with the Internet of Medical Things and artificial intelligence tools to provide real-time data and point-of-location diagnoses [6]. This can help policymakers make effective and time-appropriate decisions to contain the spread of a pandemic like COVID-19 [7].

The main domains where nanomedicine is assisting in the development of vaccines for COVID-19 are peptide/protein or DNA/mRNA encapsulation, multivalent peptide/protein display, mimicking of the multivalent features of the pathogen and quick reaction of NPs with APCs [3]. It is no secret that nanomaterials have immunomodulatory properties. With that in mind, the concept of ‘nanoimmunity by design’ is gaining currency [8]. This is particularly relevant for COVID-19 with regard to targeting the body's immune response to the coronavirus.

Nanomaterials, including dendrimers, liposomes, polymeric nanomaterials, various organic and inorganic and hybrid NPs and many other self-assembled nanostructures, are being explored for their possible role as delivery vehicles/antiviral agents against many diseases, and COVID-19 is no exception [9,10]. Around nine nanovaccine candidates are currently being developed (in Phase 1 and preclinical trials) against SARS-CoV-2 [11]. Nanomaterials not only aid in the design of novel vaccines against coronavirus, but they also enhance the efficacy of repurposed drugs. They aid in the sustained release of vaccine-related components, such as peptides, mRNA, RNAi and antigens, that are attached via conjugation or encapsulation and prevent their degradation and increase their bioavailability and also act as a carrier for vaccine components and help maintain immune homeostasis. For instance, lipid-based NPs in conjunction with peptides that can penetrate cells are being explored for the delivery of vaccines and drugs against SARS-CoV-2. This is because they are highly stable, show sustained release, have high bioavailability and are highly permeable across cell membranes. The most promising vaccine, mRNA-1273, is loaded onto lipid NPs; the BNT162 vaccine also uses lipid NPs for encapsulation [12]. Angiotensin-converting enzyme inhibitor-containing, remdesivir-loaded PLGA NPs have also been reported in a significant study [13]. In addition, researchers are exploring the potential use of ARCT-021, which utilizes lipid-enabled and unlocked nucleic acid-modified RNA NPs for vaccine delivery [14]. Coronavirus-like particles termed ‘nanoinhibitors’ are also being explored as possible vaccine candidates for SARS-CoV-2 [15]. Recent reports suggest the possibility of using the intranasal route for immunization against coronavirus. In addition, gold NP-adjuvanted S proteins are being explored as a possible vaccine candidate, though they have not proven to be very efficacious. Nanomedicine is thus helping a great deal in developing noninvasive approaches for immunizing against COVID-19.

Several studies point to enhancement of the efficacy of antiviral drugs against COVID-19 when given as a nanoformulation [2,13]. Another area of nanomedicine research that is also touted as a supplementary therapeutic strategy for managing COVID-19 is the design of food and drugs that can target bad bacteria in the gut. For instance, the plasma membrane of the human cell was utilized to construct nanosponges that aid in neutralizing SARS-CoV-2 after binding with it [16].

Inorganic nanomaterials like graphene oxide (GO) sheets with AgNPs, cationic carbon dots and Ag_2_S nanoclusters (NCs) can possibly be used to inhibit the replication and proliferation of as well as infection with SARS-CoV-2 [17]. Metal NPs such as Ag and Cu and CuO NPs act as excellent antiviral agents, and it is proposed that they can interact with the proteins present on the surface of the virus, inhibiting replication of the SARS-CoV-2 virus. Because of this, these metallic NPs can be used for disinfection purposes. Graphene-derived NPs such as graphene oxide (GO), sulfated GO and reduced GO sheets as well as photocatalytic NPs like TiO_2_ and polymersomes (polymer-like liposomes) are also being explored for their inhibitory effects on the SARS-CoV-2 virus [18,19].

Mesenchymal stem cell transplantation is showing promise in improving the outcomes of patients infected with SARS-CoV-2. Exosomes derived from mesenchymal stem cells are considered excellent nanovehicles for drugs targeting COVID-19, as they exhibit good biocompatibility and safety in comparison with synthetic nanovesicles [20].

Another area of research that is presently in the nascent stage is the development of textile materials for face masks that can trap and then eradicate the SARS-CoV-2 virus [21]. This may help in preventing the spread of COVID-19 via the nasal route. An interesting dimension that is being explored is the production of vaccines on edible plant tissues, as plant viruses and bacteriophages are proving to be stable nanocarriers, showing stability under gastrointestinal conditions [22]. This is especially relevant, as SARS-CoV-2 is a zoonotic virus, and this can help vaccinate livestock too. Also worth mentioning is nanotechnology-based microneedle vaccine delivery, which helps improve patient compliance with vaccination, as it is pain-free [23]. This technique has been attempted with COVID-19, and the initial results are encouraging; however, a lot needs to be explored before finalizing it for clinical applications.

Another interesting way in which nanotechnology can help us in our battle against coronavirus is by assisting in the design of novel materials that can be utilized to make effective personal protective equipment and face masks. In addition, sanitizers and surface sprays that can effectively disinfect and sanitize our living spaces with decreased toxicity can be developed using nanotechnology [11].

One wonders why, despite the many benefits of nanomedicine, translating nanoproducts from bench to bedside remains challenging, especially with regard to COVID-19. Nanomedicine has attracted attention from researchers across the globe, but there are certain roadblocks to its conversion to commercial products. The companies involved in the production of nanomedical drugs and devices face the challenge of higher production costs with nanodrugs compared with traditional drugs. In addition, for hospitals, the cost of acquiring these medicines is very high, leading to reluctance on the part of the healthcare industry to use these drugs. More cost–effectiveness studies of nanomedicine need to be done to convince the profit-driven healthcare and insurance industry of the importance of this approach and influence decision-making at the policy level [24].

However, with respect to COVID-19, and in general, the most important challenge that needs to be taken care of is the regulatory aspect of nanomedicine. The properties of drugs based on nanotechnology change at the nanoscale, and this alters their biosafety profile. At the nanoscale, the distinction between a medical device and a medicinal product becomes blurred, so regulation becomes more difficult. This is also compounded by the dearth of data and lack of standardization of testing protocols. One solution could be designing product-focused, science-based regulatory policy that gives weight to variances in legal standards for diverse product classes. In addition, premarket review (i.e., submission of data regarding the efficacy and regulatory status of a nanotechnology-based product by the applicant) should be made compulsory. There is an urgency with respect to the design of vaccines for COVID-19 that makes following any kind of regulatory framework even harder.

Thus, one can conclude that nanomedicine is helping to combat the challenges associated with SARS-CoV-2 vaccine design and development; however, for this to be successful, a very strong network must be established between academia and industry. Fortunately, things have moved in the right direction, and excellent vaccine candidates have emerged from public–private collaborations. However, one needs to be cautious with respect to the regulatory challenges of nanomedicine, and suitable checks and balances, including risk assessments, are needed.
